# A Dunkl type generalization of Szász operators via post-quantum calculus

**DOI:** 10.1186/s13660-018-1878-5

**Published:** 2018-10-22

**Authors:** Abdullah Alotaibi, Md. Nasiruzzaman, M. Mursaleen

**Affiliations:** 1 Operator Theory and Applications Research Group, Department of Mathematics, Faculty of Science, King Abdulaziz University, Jeddah, Saudi Arabia; 2 Department of Computer Science (SEST), Jamia Hamdard, New Delhi, India; 3 Department of Mathematics, Aligarh Muslim University, Aligarh, India

**Keywords:** 41A25, 41A36, 33C45, $(p,q)$-integers, $(p,q)$-analogues of the exponential function, Dunkl analogue, Szász operator, modulus of continuity

## Abstract

The object of this paper to construct Dunkl type Szász operators via post-quantum calculus. We obtain some approximation results for these new operators and compute convergence of the operators by using the modulus of continuity. Furthermore, we obtain the rate of convergence of these operators for functions belonging to the Lipschitz class. We also study the bivariate version of these operators.

## Introduction and preliminaries

The first well-known positive linear operator introduced by S.N. Bernstein [9] in 1912 plays an important role in approximation theory, and the first *q*-analogue of the well-known Bernstein polynomials was introduced by Lupaş [16] in 1987 who applied the idea of *q*-integers. In 1997, Phillips considered another *q*-analogue of the classical Bernstein polynomials [26]. Later on, many authors introduced *q*-generalization of various operators and investigated several approximation properties. In 1950, for $x\geq0$ and $f\in C[0,\infty )$, a positive linear operator was introduced by Szász [30].

A $(p,q)$-integer $[n]_{p,q}$ is defined as $[n]_{p,q}:=\frac {p^{n}-q^{n}}{p-q}, n=0,1,2,\dots, 0< q< p\leq1$. Recently, Mursaleen et al. [19] applied the $(p,q)$-calculus in the field approximation theory and introduced the first $(p,q)$-analogue of Bernstein operators as

1.1 $$B_{m,\ell }^{p,q}(f;x)=\frac{1}{p^{\frac{n(n-1)}{2}}}\sum_{k=0}^{n}{\left[\begin{array}{c} n\\ k \end{array}\right]}_{p,q}p^{\frac{k(k-1)}{2}}x^{k}\prod_{s=0}^{n-k-1}\left(p^{s}-q^{s}x\right)f\left(\frac{{\left[k\right]}_{p,q}}{p^{k-n}{\left[n\right]}_{p,q}}\right),\;x\in [0,1].$$

They have also investigated several approximation properties by defining different positive linear operators in an approximation process based on a $(p,q)$-analogue (see [1–8, 14, 18, 23–25]). Recently they have also studied the Szász-type operators via Dunkl generalizations (see [17, 20–22, 28]). After that many authors introduced *q*-generalizations of various operators and published their work on Dunkl type generalization (see [10, 13, 29, 31]).

There are two types of a $(p,q)$-analogue of the exponential function (see [15]),

$$ e_{p,q}(x)=\sum_{n=0}^{\infty}p^{\frac{n(n-1)}{2}} \frac {x^{n}}{[n]_{p,q}!} \quad\text{and}\quad E_{p,q}(x)=\sum_{n=0}^{\infty}q^{\frac {n(n-1)}{2}} \frac{x^{n}}{[n]_{p,q}!}, $$

which satisfy the equality $e_{p,q}(x)E_{p,q}(-x)=1$, and for $p=1$, $e_{p,q}(x)$ and $E_{p,q}(x)$ reduce to *q*-exponential functions. For $x\geq 0, f\in C[0,\infty),\mu\geq0, n\in\mathbb{N}$, Sucu [29] defined a Dunkl analogue of Szász operators via a generalization of the exponential function given by [27]. Ben Cheikh et al. [10] introduced the *q*-Dunkl classical *q*-Hermite type polynomials. They gave definitions of *q*-Dunkl analogues of exponential functions, recursion relations and notations for $\mu>-\frac{1}{2}$ and $0< q<1$. For $\mu >\frac{1}{2}, x\geq0, 0< q<1$ and $f\in C[0,\infty)$, Gürhan Içöz gave a Dunkl generalization of Szász operators via *q*-calculus [12] as

1.2 $$ D_{n,q}(f;x)=\frac{1}{e_{\mu,q}([n]_{q}x)}\sum_{k=0}^{\infty} \frac{([n]_{q}x)^{k}}{\gamma_{\mu,q}(k)}f \biggl( \frac{1-q^{2\mu\theta _{k}+k}}{1-q^{n}} \biggr). $$

Here we define $(p,q)$-Dunkl analogues of exponential functions, recursion relations and notations for $\mu>\frac{1}{2}$ and $0< q< p\leq1$, respectively, by

1.3 $$ e_{\mu,p,q}(x)=\sum_{n=0}^{\infty}p^{\frac{n(n-1)}{2}} \frac {x^{n}}{\gamma _{\mu,p,q}(n)}, \quad x\in{}[0,\infty), n\in\mathbb{N}\ (\text{natural number}). $$

We define an explicit formula for $\gamma_{\mu,p,q}(n)$ as follows:

1.4 $$\begin{aligned} &\gamma_{\mu,p,q}(n) \\ &\quad=\frac{\prod_{i=0}^{[\frac{n+1}{2}]-1}p^{2\mu (-1)^{i+1}+1} ( (p^{2})^{i}p^{2\mu+1}-(q^{2})^{i}q^{2\mu+1} ) \prod_{j=0}^{[\frac{n}{2}]-1}p^{2\mu(-1)^{j}+1} ( (p^{2})^{j}p^{2}-(q^{2})^{j}q^{2} ) }{(p-q)^{n}}, \end{aligned}$$

where $[\frac{n+1}{2}]$ and $[\frac{n}{2}]$ denote the greatest integer functions for $n\in\mathbb{N}\cup\{0\}$, and we have

$$ (x-y)_{p,q}^{n}=\textstyle\begin{cases} \prod_{i=0}^{n-1}(p^{i}x-q^{i}y) & \text{if }n\in\mathbb{N,} \\ 1 & \text{if }n=0.\end{cases} $$

Also

1.5 $$ \begin{aligned} &\gamma_{\mu,p,q}(n+1)= \frac{p^{2\mu(-1)^{n+1}+1}({p^{2\mu \theta_{n+1}+n+1}-q^{2\mu\theta_{n+1}+n+1}})}{(p-q)} \gamma_{\mu,p,q}(n),\quad n \in\mathbb{N},\\ & \theta_{n}=\textstyle\begin{cases} 0 & \text{if }n=2m, m\in\mathbb{N}, \\ 1 & \text{if }n=2m+1, m\in\mathbb{N}.\end{cases}\displaystyle \end{aligned} $$

We can derive some of the special cases of $\gamma_{\mu,p,q}(n)$ as follows:

$$\begin{aligned} &\gamma_{\mu,p,q}(0)=1\ \bigl(\text{from } (p,q)\text{-binomial expansion} \bigr), \\ &\gamma_{\mu,p,q}(1)=p^{-2\mu+1} \biggl(\frac{p^{2\mu+1}-q^{2\mu+1}}{p-q} \biggr), \\ &\gamma_{\mu,p,q}(2)=p^{2} \biggl(\frac{p^{2\mu+1}-q^{2\mu+1}}{p-q} \biggr) \biggl(\frac{p^{2}-q^{2}}{p-q} \biggr), \\ &\gamma_{\mu,p,q}(3)=p^{3-2\mu} \biggl(\frac{p^{2\mu+1}-q^{2\mu+1}}{p-q} \biggr) \biggl(\frac{p^{2}-q^{2}}{p-q} \biggr) \biggl(\frac{p^{2\mu+3}-q^{2\mu +3}}{p-q} \biggr), \\ &\gamma_{\mu,p,q}(4)=p^{4} \biggl(\frac{p^{2\mu+1}-q^{2\mu+1}}{p-q} \biggr) \biggl(\frac{p^{2}-q^{2}}{p-q} \biggr) \biggl(\frac{p^{2\mu+3}-q^{2\mu+3}}{p-q} \biggr) \biggl(\frac{p^{4}-q^{4}}{p-q} \biggr), \\ &\gamma_{\mu,p,q}(5)=p^{5-2\mu} \biggl( \frac{p^{2\mu+1}-q^{2\mu +1}}{p-q} \biggr) \biggl( \frac{p^{2}-q^{2}}{p-q} \biggr) \biggl( \frac{p^{2\mu +3}-q^{2\mu+3}}{p-q} \biggr)\\ &\phantom{\gamma_{\mu,p,q}(5)=}{}\times \biggl( \frac{p^{4}-q^{4}}{p-q} \biggr) \biggl( \frac{p^{2\mu+5}-q^{2\mu+5}}{p-q} \biggr). \end{aligned}$$

## Auxiliary results

For any $x\in[0,\infty), f \in C[0,\infty) n \in\mathbb{N}, 0< q< p\leq1$, and $\mu>\frac{1}{2}$, we define

2.1 $$ D_{n,p,q}(f;x)=\frac{1}{e_{\mu,p,q}([n]_{p,q}x)}\sum _{k=0}^{\infty}\frac{ ([n]_{p,q}x)^{k}}{\gamma_{\mu,p,q}(k)}p^{\frac{k(k-1)}{2}}f \biggl( \frac{p^{2\mu\theta_{k}+k}-q^{2\mu\theta_{k}+k}}{p^{k-1}(p^{n}-q^{n})} \biggr). $$

If $p = 1$ in (2.1), then the Dunkl generalization of Szász operators via $(q)$-calculus (1.2) becomes a particular case of $(p, q) $-operators defined by us. Thus we can say that our operators can be considered a generalization of operators (1.2).

### Lemma 2.1

*Let* $D_{n,p,q}(\cdot,\cdot)$ *be the operators given by* (2.1). *Then we have the following results*:

1. $D_{n,p,q}(e_{0};x)=1$.
2. $D_{n,p,q}(e_{1};x)=x$.
3. $x^{2}+\frac{q^{2\mu}}{[n]_{p,q}}[1-2 \mu]_{p,q}\frac{e_{\mu,p,q}(\frac{q}{p}[n]_{p,q}x)}{e_{\mu,p,q}([n]_{p,q}x)} x\leq D_{n,p,q}(e_{2};x)\leq x^{2}+\frac{1}{[n]_{p,q}}[1+2\mu]_{p,q}x$,

*where* $e_{j}(t)=t^{j}, j=0,1,2, \ldots$ .

### Proof

(1) $D_{n,p,q}(1;x)=\frac{1}{e_{\mu,p,q}([n]_{p,q}x)}\sum_{k=0}^{\infty}\frac{([n]_{p,q}x)^{k}}{\gamma_{\mu,p,q}(k)}p^{\frac{k(k-1)}{2}}=1\ (\mbox{from (1.3)})$.

(2)

$$\begin{aligned} D_{n,p,q}(e_{1};x)&= \frac{1}{e_{\mu,p,q}([n]_{p,q}x)}\sum _{k=0}^{\infty}\frac{([n]_{p,q}x)^{k}}{\gamma_{\mu,p,q}(k)}p^{\frac{k(k-1)}{2}} \biggl( \frac{p^{2\mu\theta_{k}+k}-q^{2\mu\theta_{k}+k}}{p^{k-1}(p^{n}-q^{n})} \biggr) \\ &= \frac{1}{[n]_{p,q}}\frac{1}{e_{\mu,p,q}([n]_{p,q}x)}\sum_{k=1}^{\infty}\frac{([n]_{p,q}x)^{k}}{\gamma_{\mu,p,q}(k-1)}p^{\frac{(k-1)(k-2)}{2}} \\ &= \frac{1}{[n]_{p,q}}\frac{1}{e_{\mu,p,q}([n]_{p,q}x)}\sum_{k=0}^{\infty}\frac{([n]_{p,q}x)^{k+1}}{\gamma_{\mu,p,q}(k)}p^{\frac{k(k-1)}{2}} \\ &= \frac{x}{e_{\mu,p,q}([n]_{p,q}x)}\sum_{k=0}^{\infty}\frac {([n]_{p,q}x)^{k}}{\gamma_{\mu,p,q}(k)}p^{\frac{k(k-1)}{2}} \\ &= x. \end{aligned}$$

(3)

$$\begin{aligned} D_{n,p,q}(e_{2};x)&=\frac{1}{e_{\mu,p,q}([n]_{p,q}x)}\sum _{k=0}^{\infty}\frac{([n]_{p,q}x)^{k}}{\gamma_{\mu,p,q}(k)}p^{\frac{k(k-1)}{2}} \biggl( \frac{p^{2\mu\theta_{k}+k}-q^{2\mu\theta_{k}+k}}{p^{k-1}(p^{n}-q^{n})} \biggr)^{2} \\ &=\frac{1}{[n]_{p,q}^{2}}\frac{1}{e_{\mu,p,q}([n]_{p,q}x)}\sum_{k=1}^{\infty}\frac{([n]_{p,q}x)^{k}}{\gamma_{\mu,p,q}(k-1)}p^{\frac{k^{2}-5k+4}{2}} \biggl( \frac{p^{2\mu\theta_{k}+k}-q^{2\mu\theta_{k}+k}}{(p-q)} \biggr) \\ &=\frac{1}{[n]_{p,q}^{2}}\frac{1}{e_{\mu,p,q}([n]_{p,q}x)}\sum_{k=1}^{\infty}\frac{([n]_{p,q}x)^{k}}{\gamma_{\mu,p,q}(k-1)}p^{\frac{(k-1)(k-4)}{2}} \biggl( \frac{p^{2\mu\theta_{k}+k}-q^{2\mu\theta_{k}+k}}{(p-q)} \biggr), \end{aligned}$$

hence

2.2 $$\begin{aligned} & D_{n,p,q}(e_{2};x) \\ &\quad=\frac{1}{[n]_{p,q}^{2}} \frac{1}{e_{\mu,p,q}([n]_{p,q}x)}\sum_{k=0}^{\infty}\frac{([n]_{p,q}x)^{k+1}}{\gamma_{\mu,p,q}(k)}p^{\frac {k(k-3)}{2}} \biggl( \frac{p^{2\mu\theta_{k+1}+k+1}-q^{2\mu\theta _{k+1}+k+1}}{(p-q)} \biggr). \end{aligned}$$

From a simple calculation we know that

2.3 $$ [2\mu\theta_{k+1}+k+1]_{p,q}=p^{2\mu(-1)^{k}+1}[2 \mu \theta_{k}+k]_{p,q}+q^{2\mu\theta_{k}+k}\bigl[2 \mu(-1)^{k}+1\bigr]_{p,q}. $$

For $k=2k$, Eq. (2.3) implies

2.4 $$ [2\mu\theta_{2k+1}+2k+1]_{p,q}=p^{2\mu+1} \biggl(\frac{p^{2\mu \theta_{2k}+2k}-q^{2\mu\theta_{2k}+2k}}{p-q} \biggr)+q^{2\mu \theta_{2k}+2k}[1+2\mu]_{p,q}, $$

and for $k=2k+1$, we have

2.5 $$\begin{aligned}{} [2\mu\theta_{2k+2}+2k+2]_{p,q}={}&p^{-2\mu+1} \biggl(\frac{p^{2\mu \theta_{2k+1}+2k+1}-q^{2\mu\theta_{2k+1}+2k+1}}{p-q} \biggr) \\ &{}+q^{2\mu \theta_{2k+1}+2k+1}[1-2\mu]_{p,q}. \end{aligned}$$

Now by separating (2.2), into the even and odd terms and using (2.4)–(2.5), we have

$$\begin{aligned} & D_{n,p,q}(e_{2};x) \\ &\quad =\frac{1}{[n]_{p,q}^{2}}\frac{1}{e_{\mu,p,q}([n]_{p,q}x)}\sum_{k=0}^{\infty}p^{2\mu(-1)^{k}+1} \frac{([n]_{p,q}x)^{k+1}}{\gamma_{\mu,p,q}(k)}p^{\frac {k(k-3)}{2}} \biggl( \frac{p^{2\mu\theta_{k}+k}-q^{2\mu\theta _{k}+k}}{(p-q)} \biggr)\Big\vert _{k=2k,2k+1} \\ &\qquad{}+\frac{1}{[n]_{p,q}^{2}}\frac{1}{e_{\mu,p,q}([n]_{p,q}x)}\sum_{k=0}^{\infty}\frac{([n]_{p,q}x)^{2k+1}}{\gamma_{\mu,p,q}(2k)}p^{k(2k-3)} q^{2\mu\theta_{2k}+2k}[1+2\mu]_{p,q} \\ &\qquad{}+\frac{1}{[n]_{p,q}^{2}}\frac{1}{e_{\mu,p,q}([n]_{p,q}x)}\sum_{k=0}^{\infty}\frac{([n]_{p,q}x)^{2k+2}}{\gamma_{\mu,p,q}(2k+1)}p^{(k-1)(2k+1)} q^{2\mu\theta_{2k+1}+2k+1}[1-2\mu]_{p,q} \\ &\quad\geq\frac{x^{2}}{e_{\mu,p,q}([n]_{p,q}x)}\sum_{k=0}^{\infty}p^{2\mu (-1)^{k+1}} \frac{([n]_{p,q}x)^{k}}{\gamma_{\mu,p,q}(k)}p^{\frac{k(k-1)}{2}} \\ &\qquad{}+\frac{1}{q[n]_{p,q}^{2}}\frac{1}{e_{\mu,p,q}([n]_{p,q}x)}[1-2 \mu]_{p,q}\sum _{k=0}^{\infty}\frac{(q[n]_{p,q}x)^{2k+1}}{\gamma_{\mu ,q}(2k)}p^{k(2k-3)} \\ &\qquad{}+\frac{q^{2\mu-1}}{[n]_{p,q}^{2}}\frac{1}{e_{\mu,p,q}([n]_{p,q}x)}[1-2\mu]_{p,q}\sum _{k=0}^{\infty}\frac{(q[n]_{p,q}x)^{2k+2}}{\gamma_{\mu,p,q}(2k+1)}p^{(k-1)(2k+1)}. \end{aligned}$$

Here we have used the inequality $[1-2\mu]_{p,q} \leq[1+2\mu]_{p,q}$, and, for $0< q< p\leq1$ and $\mu> \frac{1}{2}$, a simple calculation led to $p^{2\mu}\leq1, p^{-2\mu}\geq1$. Therefore,

$$\begin{aligned} D_{n,p,q}(e_{2};x) &\geq x^{2}+\frac{q^{2\mu}}{[n]_{p,q}} \frac{x}{e_{\mu,p,q}([n]_{p,q}x)}[1-2 \mu]_{p,q}\sum_{k=0}^{\infty}\frac{(\frac {q}{p}[n]_{p,q}x)^{k}}{\gamma_{\mu,p,q}(k)}p^{\frac{k(k-1)}{2}} \\ &\geq x^{2}+\frac{q^{2\mu}}{[n]_{p,q}}[1-2 \mu]_{p,q} \frac{e_{\mu ,p,q}(\frac{q}{p}[n]_{p,q}x)}{e_{\mu,p,q}([n]_{p,q}x)} x. \end{aligned}$$

On the other hand, we have

$$\begin{aligned} &D_{n,p,q}(e_{2};x)\\ &\quad\leq x^{2}+\frac{1}{[n]_{p,q}} \frac{x}{e_{\mu,p,q}([n]_{p,q}x)}[1+2 \mu]_{p,q}\sum_{k=0}^{\infty}\frac{(q[n]_{p,q}x)^{2k}}{\gamma_{\mu,q}(2k)}p^{k(2k-3)} \\ &\qquad{}+\frac{q^{2\mu}}{[n]_{p,q}}\frac{x}{e_{\mu,p,q}([n]_{p,q}x)}[1+2\mu]_{p,q}\sum _{k=0}^{\infty}\frac{(q[n]_{p,q}x)^{2k+1}}{\gamma_{\mu,p,q}(2k+1)}p^{(k-1)(2k+1)} \\ &\quad\leq x^{2}+\frac{1}{[n]_{p,q}}\frac{x}{e_{\mu,p,q}([n]_{p,q}x)}[1+2 \mu]_{p,q}\sum_{k=0}^{\infty}\frac{(\frac{q}{p}[n]_{p,q}x)^{2k}}{\gamma_{\mu,q}(2k)}p^{k(2k-1)} \\ &\qquad{}+\frac{q^{2\mu}}{[n]_{p,q}}\frac{x}{e_{\mu,p,q}([n]_{p,q}x)}[1+2\mu]_{p,q}\sum _{k=0}^{\infty}\frac{(\frac{q}{p}[n]_{p,q}x)^{2k+1}}{\gamma_{\mu,p,q}(2k+1)}p^{k(2k+1)} \\ &\quad\leq x^{2}+\frac{1}{[n]_{p,q}}\frac{x}{e_{\mu,p,q}([n]_{p,q}x)}[1+2 \mu]_{p,q}\sum_{k=0}^{\infty}\frac{([n]_{p,q}x)^{k}}{\gamma_{\mu ,p,q}(k)}p^{\frac{k(k-1)}{2}} \\ &\quad\leq x^{2}+\frac{1}{[n]_{p,q}}[1+2 \mu]_{p,q}x. \end{aligned}$$

 □

### Lemma 2.2

*Let the operators* $D_{n,p,q}(\cdot,\cdot)$ *be given by* (2.1). *Then we have the following results*:

1. $D_{n,p,q}(e_{1}-x;x)=0$.
2. $\frac{q^{2\mu}}{[n]_{p,q}}[1-2 \mu]_{p,q}\frac{e_{\mu,p,q}(\frac{q}{p}[n]_{p,q}x)}{e_{\mu,p,q}([n]_{p,q}x)} x\leq D_{n,p,q}((e_{1}-x)^{2};x)\leq\frac{1}{[n]_{p,q}}[1+2\mu]_{p,q}x$.

## Main results

In this section we obtain a Korovkin’s type approximation theorem and compute convergence of the considered operators by using the modulus of continuity and also rate of convergence for functions belonging to the Lipschitz class presented here.

Let $C_{B}(\mathbb{R^{+}})$ be the set of all bounded and continuous functions on $\mathbb{R^{+}}=[0,\infty)$, which is a linear normed space with

$$ \Vert f \Vert _{C_{B}}=\sup_{x\geq0} \bigl\vert f(x) \bigr\vert . $$

And also let

$$ H:=\biggl\{ f:x \in[0,\infty), \frac{f(x)}{1+x^{2}} \mbox{ is convergent as } x \to\infty\biggr\} . $$

In order to obtain the convergence results for the operators $D_{n,p,q}(\cdot,\cdot)$, we take $q=q_{n}, p=p_{n}$ where $q_{n}\in (0,1)$ and $p_{n}\in(q_{n},1]$ satisfy

3.1 $$\begin{aligned} \lim_{n}p_{n} \to1,\qquad \lim _{n}q_{n} \to1 \quad\mbox{and}\quad \lim _{n}p_{n}^{n} \to a,\qquad \lim _{n}q_{n}^{n} \to b. \end{aligned}$$

### Theorem 3.1

*Let* $p=p_{n}, q=q_{n}$ *satisfy* (3.1), *with* $0< q_{n}< p_{n}\leq 1$. *Let* $D_{n,p_{n},q_{n}}(\cdot,\cdot)$ *be the operators given by* (2.1). *Then for any function* $f \in X[0,\infty) \cap H$,

$$ \lim_{n\to\infty}D_{n,p_{n},q_{n}}(f;x)=f(x) $$

*uniformly on each compact subset of* $[0,\infty)$.

### Proof

The proof is based on the well-known Korovkin’s theorem regarding the convergence of a sequence of linear and positive operators, so it is enough to prove the conditions

$$ \lim_{n\to\infty}{D}_{n,p_{n},q_{n}}(e_{j};x)=x^{j},\quad j=0,1,2, $$

uniformly on $[0,1]$. Clearly, from (3.1) and $\frac{1}{[n]_{p_{n},q_{n}}} \to0$, when $n \to \infty$, we have

$$ \lim_{n \to\infty}{D}_{n,p_{n},q_{n}}(e_{1};x)=x,\qquad \lim _{n \to\infty}{D}_{n,p_{n},q_{n}}(e_{2};x)=x^{2}, $$

which completes the proof. □

We recall the weighted spaces of the functions on $\mathbb{R}^{+}$, which are defined as follows:

$$\begin{aligned} &P_{\rho}\bigl(\mathbb{R}^{+}\bigr) = \bigl\{ f: \bigl\vert f(x) \bigr\vert \leq M_{f}\rho(x) \bigr\} , \\ &Q_{\rho}\bigl(\mathbb{R}^{+}\bigr) = \bigl\{ f: f \in P_{\rho}\bigl(\mathbb{R}^{+}\bigr) \cap C[0,\infty) \bigr\} , \\ &Q^{k}_{\rho}\bigl(\mathbb{R}^{+}\bigr) = \biggl\{ f: f \in Q_{\rho}\bigl(\mathbb{R}^{+}\bigr) \mbox{ and } \lim _{x \to\infty}\frac{f(x)}{\rho(x)} =k\ (k \mbox{ is a constant}) \biggr\} , \end{aligned}$$

where $\rho(x)=1+x^{2}$ is a weight function and $M_{f}$ is a constant depending only on *f*. And $Q_{\rho}(\mathbb{R}^{+})$ is a normed space with the norm $\Vert f \Vert _{\rho}=\sup_{x \geq0} \frac{ \vert f(x) \vert }{\rho(x)}$.

### Theorem 3.2

*Let* $p=p_{n}, q=q_{n}$ *satisfy* (3.1) *with* $0< q_{n}< p_{n}\leq1$ *and let* $D_{n,p_{n},q_{n}}(\cdot,\cdot)$ *be the operators given by* (2.1). *Then for any function* $f\in Q_{\rho}^{k}(\mathbb {R}^{+})$ *we have*

$$ \lim_{n\rightarrow\infty} \bigl\Vert D_{n,p_{n},q_{n}}(f;x)-f \bigr\Vert _{\rho}=0. $$

### Proof

From Lemma 2.1, the first condition of (1) is fulfilled for $\tau=0$. Now for $\tau=1,2$, it is easy to see that from (2)–(3) of Lemma 2.1, by using (3.1),

$$ \lim_{n\rightarrow\infty}\bigl\Vert D_{n,p_{n},q_{n}} \bigl( e_{1}^{\tau};x \bigr) -x^{\tau } \bigr\Vert _{\rho}=0. $$

This completes the proof**.** □

Here we calculate the rate of convergence of operators (2.1) by means of modulus of continuity and Lipschitz type maximal functions.

Let $f \in C[0,\infty] $. The modulus of continuity of *f*, denoted by $\omega(f,\delta)$, gives the maximum oscillation of *f* in any interval of length not exceeding $\delta>0$ and it is given by the relation

3.2 $$ \omega(f,\delta)=\sup_{ \vert y-x \vert \leq\delta} \bigl\vert f(y)-f(x) \bigr\vert , \quad x,y \in[0,\infty). $$

It is known that $\lim_{\delta\to0+}\omega(f,\delta)=0$ for $f \in C[0, \infty)$, and for any $\delta>0$ one has

3.3 $$ \bigl\vert f(y)- f(x) \bigr\vert \leq \biggl( \frac{ \vert y-x \vert }{\delta}+1 \biggr)\omega(f,\delta). $$

### Theorem 3.3

*Let* $f\in\tilde{C}[0,\infty), x \in[0,\infty)$. *Then for* $0< q< p\leq1$ *the operators* $D_{n,p,q}(\cdot,\cdot)$ *defined by* (2.1) *satisfy*

$$ \bigl\vert D_{n,p,q}(f;x)-f(x) \bigr\vert \leq \bigl\{ \sqrt{ [1+2\mu]_{p,q}x} \bigr\} \omega \biggl(f; \frac{1}{\sqrt{[n]_{p,q}}} \biggr), $$

*where* $\tilde{C}[0,\infty)$ *is the space of uniformly continuous functions on* $\mathbb{R}^{+}$ *and* $\omega(f,\delta)$ *is the modulus of continuity of a function* $f \in\tilde{C}[0,\infty)$ *defined in* (3.2).

### Proof

We prove the claim by using (3.2)–(3.3) and Cauchy–Schwarz inequality:

$$\begin{aligned} & \bigl\vert D_{n,p,q}(f;x)-f(x) \bigr\vert \\ &\quad \leq \frac{1}{e_{\mu,p,q}([n]_{p,q}x)}\sum_{k=0}^{\infty}\frac{([n]_{p,q}x)^{k}}{\gamma_{\mu,p,q}(k)}p^{\frac{k(k-1)}{2}} \biggl\vert f \biggl( \frac{p^{2\mu\theta_{k}+k}-q^{2\mu\theta_{k}+k}}{p^{k-1}(p^{n}-q^{n})} \biggr) -f(x) \biggr\vert \\ &\quad\leq \frac{1}{e_{\mu,p,q}([n]_{p,q}x)}\sum_{k=0}^{\infty}\frac{([n]_{p,q}x)^{k}}{\gamma_{\mu,p,q}(k)}p^{\frac{k(k-1)}{2}} \biggl\{ 1+ \frac{1}{\delta} \biggl\vert \biggl( \frac{p^{2\mu\theta_{k}+k}-q^{2\mu\theta_{k}+k}}{p^{k-1}(p^{n}-q^{n})} \biggr) -x \biggr\vert \biggr\} \omega(f;\delta) \\ &\quad= \Biggl\{ 1+\frac{1}{\delta} \Biggl( \frac{1}{e_{\mu,p,q}([n]_{p,q}x)}\sum _{k=0}^{\infty}\frac{([n]_{p,q}x)^{k}}{\gamma_{\mu,p,q}(k)}p^{\frac {k(k-1)}{2}} \biggl\vert \frac{p^{2\mu\theta_{k}+k}-q^{2\mu\theta _{k}+k}}{p^{k-1}(p^{n}-q^{n})}-x \biggr\vert \Biggr) \Biggr\} \omega(f;\delta) \\ &\quad \leq \Biggl\{ 1+\frac{1}{\delta} \Biggl( \frac{1}{e_{\mu,p,q}([n]_{p,q}x)} \sum _{k=0}^{\infty}\frac{([n]_{p,q}x)^{k}}{\gamma_{\mu,p,q}(k)}p^{\frac {k(k-1)}{2}} \biggl( \frac{p^{2\mu\theta_{k}+k}-q^{2\mu\theta _{k}+k}}{p^{k-1}(p^{n}-q^{n})}-x \biggr)^{2} \Biggr)^{\frac{1}{2}} \\ &\qquad{}\times\bigl(D_{n,p,q}(e_{0};x) \bigr)^{\frac {1}{2}} \Biggr\} \omega(f;\delta) \\ &\quad = \biggl\{ 1+\frac{1}{\delta} \bigl(D_{n,p,q}(e_{1}-x)^{2};x \bigr)^{\frac {1}{2}} \biggr\} \omega(f;\delta) \\ &\quad\leq\biggl\{ 1+\frac{1}{\delta} \sqrt{\frac{1}{[n]_{p,q}}[1+2\mu ]_{p,q}x} \biggr\} \omega(f;\delta), \end{aligned}$$

where, choosing $\delta=\delta_{n,p,q}=\sqrt{\frac{1}{[n]_{p,q}}}$, we get our result. □

Now we give the rate of convergence of the operators ${D}_{n,p,q}(f;x) $ defined in (2.1) in terms of the elements of the usual Lipschitz class $\operatorname{Lip}_{M}(\nu)$.

Let $f\in C[0,\infty)$, $M>0$ and $0<\nu\leq1$. We recall that the class $\operatorname{Lip}_{M}(\nu)$ is given by

3.4 $$ \operatorname{Lip}_{M}(\nu)= \bigl\{ f: \bigl\vert f(\zeta_{1})-f( \zeta_{2}) \bigr\vert \leq M \vert \zeta_{1}- \zeta_{2} \vert ^{\nu}, \zeta_{1}, \zeta_{2}\in{}[ 0,\infty) \bigr\} . $$

### Theorem 3.4

*Let* $D_{n,p,q}(\cdot,\cdot)$ *be the operator defined in* (2.1). *Then for each* $f\in \operatorname{Lip}_{M}(\nu), M>0, 0<\nu\leq1$, *satisfying* (3.4) *we have*

$$ \bigl\vert D_{n,p,q}(f;x)-f(x) \bigr\vert \leq M \biggl( \frac{1}{[n]_{p,q}}[1+2\mu]_{p,q}x \biggr)^{\frac{\nu}{2}}. $$

### Proof

We prove the claim by using (3.4) and Hölder inequality:

$$\begin{aligned} \bigl\vert D_{n,p,q}(f;x)-f(x) \bigr\vert &\leq \bigl\vert D_{n,p,q}\bigl(f(e_{1})-f(x);x\bigr) \bigr\vert \\ &\leq D_{n,p,q} \bigl( \bigl\vert f(e_{1})-f(x) \bigr\vert ;x \bigr) \\ &\leq\vert MD_{n,p,q} \bigl( \vert e_{1}-x \vert ^{\nu};x \bigr). \end{aligned}$$

Therefore,

$$\begin{aligned} & \bigl\vert D_{n,p,q}(f;x)-f(x) \bigr\vert \\ &\quad\leq M\frac{1}{e_{\mu,p,q}([n]_{p,q}x)}\sum_{k=0}^{\infty} \frac{([n]_{p,q}x)^{k}}{\gamma_{\mu,p,q}(k)}p^{\frac{k(k-1)}{2}} \biggl\vert \frac{p^{2\mu\theta_{k}+k}-q^{2\mu\theta_{k}+k}}{p^{k-1}(p^{n}-q^{n})}-x \biggr\vert ^{\nu} \\ &\quad\leq M\frac{1}{e_{\mu,p,q}([n]_{p,q}x)}\sum_{k=0}^{\infty} \biggl( \frac{([n]_{p,q}x)^{k}p^{\frac{k(k-1)}{2}}}{\gamma_{\mu,p,q}(k)} \biggr) ^{\frac{2-\nu}{2}} \\ &\qquad{} \times \biggl( \frac{([n]_{p,q}x)^{k}p^{\frac{k(k-1)}{2}}}{\gamma_{\mu ,p,q}(k)} \biggr) ^{\frac{\nu}{2}} \biggl\vert \frac{p^{2\mu\theta _{k}+k}-q^{2\mu\theta_{k}+k}}{p^{k-1}(p^{n}-q^{n})}-x \biggr\vert ^{\nu} \\ &\quad\leq M \Biggl( \frac{1}{ ( e_{\mu,p,q}([n]_{p,q}x) ) }\sum_{k=0}^{\infty} \frac{([n]_{p,q}x)^{k}p^{\frac{k(k-1)}{2}}}{\gamma _{\mu ,p,q}(k)} \Biggr) ^{\frac{2-\nu}{2}} \\ &\qquad{} \times \Biggl( \frac{1}{ ( e_{\mu ,p,q}([n]_{p,q}x) ) }\sum_{k=0}^{\infty} \frac {([n]_{p,q}x)^{k}p^{\frac{k(k-1)}{2}}}{\gamma_{\mu,p,q}(k)} \biggl\vert \frac{p^{2\mu\theta _{k}+k}-q^{2\mu\theta_{k}+k}}{p^{k-1}(p^{n}-q^{n})}-x \biggr\vert ^{2} \Biggr) ^{\frac{\nu}{2}} \\ &\quad\leq M \bigl( D_{n,p,q}(e_{1}-x)^{2};x \bigr) ^{\frac {\nu}{2}}. \end{aligned}$$

This completes the proof. □

We denote by $C_{B}[0,\infty)$ the space of all bounded and continuous functions on $\mathbb{R}^{+}=[0,\infty)$ and

3.5 $$ C_{B}^{2}\bigl(\mathbb{R}^{+}\bigr)=\bigl\{ g \in C_{B}\bigl(\mathbb{R}^{+}\bigr):g^{\prime},g^{\prime \prime } \in C_{B}\bigl(\mathbb{R}^{+}\bigr)\bigr\} , $$

with the norm

3.6 $$ \Vert g \Vert _{C_{B}^{2}(\mathbb{R}^{+})}= \Vert g \Vert _{C_{B}(\mathbb{R}^{+})}+ \bigl\Vert g^{\prime} \bigr\Vert _{C_{B}(\mathbb{R}^{+})}+ \bigl\Vert g^{\prime\prime} \bigr\Vert _{C_{B}(\mathbb{R}^{+})}, $$

also

3.7 $$ \Vert g \Vert _{C_{B}(\mathbb{R}^{+})}=\sup_{x \in\mathbb{R}^{+}} \bigl\vert g(x) \bigr\vert . $$

### Theorem 3.5

*Let* $D_{n,p,q}(\cdot,\cdot)$ *be the operator defined in* (2.1). *Then for any* $g \in C_{B}^{2}(\mathbb{R}^{+})$ *we have*

$$ \bigl\vert D_{n,p,q}(f;x)-f(x) \bigr\vert \leq\frac{[1+2\mu]_{p,q}x}{2[n]_{p,q}} \Vert g \Vert _{C_{B}^{2}(\mathbb{R}^{+})}. $$

### Proof

Let $g \in C_{B}^{2}(\mathbb{R}^{+})$. Then by using the generalized mean value theorem in the Taylor series expansion we have

$$ g(e_{1})=g(x)+g^{\prime}(x) (e_{1}-x)+g^{\prime\prime}( \psi)\frac {(e_{1}-x)^{2}}{2}, \quad \psi\in(x,e_{1}). $$

By applying the linearity property of $D_{n,p,q}$, we have

$$ D_{n,p,q}(g,x)-g(x)=g^{\prime}(x)D_{n,p,q} \bigl((e_{1}-x);x \bigr)+\frac{g^{\prime\prime}(\psi)}{2}D_{n,p,q} \bigl((e_{1}-x)^{2};x \bigr), $$

which implies

$$ \bigl\vert D_{n,p,q}(g;x)-g(x) \bigr\vert \leq \biggl( \frac{1}{[n]_{p,q}}[1+2\mu]_{p,q}x \biggr) \frac{ \Vert g^{\prime\prime} \Vert _{C_{B}(\mathbb{R}^{+})}}{2}. $$

From (3.6) we have $\Vert g^{\prime} \Vert _{C_{B}[0,\infty)}\leq \Vert g \Vert _{C_{B}^{2}[0,\infty)}$ and so

$$ \bigl\vert D_{n,p,q}(g;x)-g(x) \bigr\vert \leq \biggl( \frac{1}{[n]_{p,q}}[1+2\mu]_{p,q}x \biggr) \frac{ \Vert g \Vert _{C_{B}^{2}(\mathbb{R}^{+})}}{2}. $$

This completes the proof due to (1) of Lemma 2.2. □

The Peetre’s *K*-functional is defined by

3.8 $$ K_{2}(f,\delta)=\inf_{C_{B}^{2}(\mathbb{R}^{+})} \bigl\{ \bigl( \Vert f-g \Vert _{C_{B}(\mathbb{R}^{+})} +\delta \bigl\Vert g^{\prime\prime } \bigr\Vert _{C_{B}^{2}(\mathbb{R}^{+})} \bigr):g\in\mathcal{W}^{2} \bigr\} , $$

where

3.9 $$ \mathcal{W}^{2}= \bigl\{ g\in C_{B}\bigl( \mathbb{R}^{+}\bigr):g^{\prime},g^{\prime \prime }\in C_{B}\bigl( \mathbb{R}^{+}\bigr) \bigr\} . $$

Then there exits a positive constant $C>0$ such that $K_{2}(f,\delta )\leq C \omega_{2}(f,\delta^{\frac{1}{2}}), \delta>0$, where the second order modulus of continuity is given by

3.10 $$ \omega_{2}\bigl(f,\delta^{\frac{1}{2}}\bigr)=\sup _{0< h< \delta^{\frac{1}{2}}}\sup_{x\in\mathbb{R}^{+}} \bigl\vert f(x+2h)-2f(x+h)+f(x) \bigr\vert . $$

### Theorem 3.6

*Let* $D_{n,p,q}(\cdot,\cdot)$ *be the operator defined in* (2.1) *and* $C_{B}[0,\infty)$ *the space of all bounded and continuous functions on* $\mathbb{R}^{+}$. *Then for* $x\in\mathbb{R}^{+}, f \in C_{B}(\mathbb{R}^{+})$ *we have*

$$ \bigl\vert D_{n,p,q}(f;x)-f(x) \bigr\vert \leq2M \biggl\{ \omega_{2} \biggl(f; \sqrt {\frac{[1+2\mu]_{p,q}x}{4[n]_{p,q}}} \biggr)+ \min \biggl(1, \frac{[1+2\mu ]_{p,q}x}{4[n]_{p,q}} \biggr) \Vert f \Vert _{C_{B}(\mathbb{R}^{+})} \biggr\} , $$

*where* *M* *is a positive constant and* $\omega_{2}(f;\delta)$ *is the second order modulus of continuity of the function* *f* *defined in* (3.10).

### Proof

We prove this claim by using Theorem (3.5):

$$\begin{aligned} &\bigl\vert D_{n,p,q}(f;x)-f(x) \bigr\vert \\ &\quad \leq \bigl\vert D_{n,p,q}(f-g;x) \bigr\vert + \bigl\vert D_{n,p,q}(g;x)-g(x) \bigr\vert + \bigl\vert f(x)-g(x) \bigr\vert \\ &\quad\leq 2 \Vert f-g \Vert _{C_{B}(\mathbb{R}^{+})}+ \frac{[1+2\mu ]_{p,q}x}{2[n]_{p,q}} \Vert g \Vert _{C_{B}^{2}(\mathbb{R}^{+})}. \end{aligned}$$

From (3.6) we clearly have $\Vert g \Vert _{C_{B}[0,\infty)}\leq \Vert g \Vert _{C_{B}^{2}[0,\infty)}$.

Therefore,

$$ \bigl\vert D_{n,p,q}(f;x)-f(x) \bigr\vert \leq2 \biggl( \Vert f-g \Vert _{C_{B}(\mathbb{R}^{+})}+\frac{[1+2\mu]_{p,q}x}{4[n]_{p,q}} \Vert g \Vert _{C_{B}^{2}(\mathbb{R}^{+})} \biggr). $$

By taking the infimum over all $g \in C_{B}^{2}(\mathbb{R}^{+})$ and using (3.8), we get

$$ \bigl\vert D_{n,p,q}(f;x)-f(x) \bigr\vert \leq2K_{2} \biggl(f;\frac{[1+2\mu]_{p,q}x}{4[n]_{p,q}} \biggr). $$

Now for an absolute constant $C>0$ given in [11] we have the relation

$$ K_{2}(f;\delta)\leq C\bigl\{ \omega_{2}(f;\sqrt{\delta})+ \min(1,\delta) \Vert f \Vert \bigr\} . $$

This completes the proof. □

## Bivariate operators and rate of convergence

In this section, we construct a bivariate extension of the operators (2.1).

Let $\mathbb{R}^{2}_{+} = [0,\infty)\times[0,\infty), f: C(\mathbb{R}^{2}_{+} )\to \mathbb{R}$ and $0< q_{n_{1}},q_{n_{2}}< p_{n_{1}},p_{n_{2}}\leq1$. We define the bivariate extension of the Dunkl $(p,q)$-Szász operators (2.1) as follows:

4.1 $$\begin{aligned} &D_{n_{1},n_{2}}^{(p_{n_{1}},p_{n_{2}}),(q_{n_{1}},q_{n_{2}})}(f;x,y) \\ &\quad =\frac{1}{e_{\mu_{1},p_{n_{1}},q_{n_{1}}}([n_{1}]_{p_{n_{1}},q_{n_{1}}}x)} \frac{1}{ e_{\mu_{2},p_{n_{2}},q_{n_{2}}}([n_{2}]_{p_{n_{2}},q_{n_{2}}}y)} \\ &\qquad{}\times \sum_{k_{1}=0}^{\infty}\sum_{k_{2}=0}^{\infty}\frac{([n_{1}]_{p_{n_{1}},q_{n_{1}}}x)^{k_{1}}}{\gamma_{\mu_{1},p_{n_{1}},q_{n_{1}}}(k_{1})} \frac {([n_{2}]_{p_{n_{2}},q_{n_{2}}}y)^{k_{2}}}{\gamma_{\mu_{2},p_{n_{2}},q_{n_{2}}}(k_{2})} \\ &\qquad{}\times p_{n_{1}}^{\frac{k_{1}(k_{1}-1)}{2}}p_{n_{2}}^{\frac{k_{2}(k_{2}-1)}{2}}f \biggl( \frac{p_{n_{1}}^{2\mu_{1}\theta_{k_{1}}+k_{1}}-q_{n_{1}}^{2\mu_{1}\theta _{k_{1}}+k_{1}}}{p_{n_{1}}^{k_{1}-1}(p_{n_{1}}^{n_{1}}-q_{n_{1}}^{n_{1}})}, \frac{p_{n_{2}}^{2\mu_{2}\theta_{k_{2}}+k_{2}}-q_{n_{2}}^{2\mu_{2}\theta_{k_{2}}+k_{2}}}{p_{n_{2}}^{k_{2}-1}(p_{n_{2}}^{n_{2}}-q_{n_{2}}^{n_{2}})} \biggr), \end{aligned}$$

where $e_{\mu_{1},p_{n_{1}},q_{n_{1}}}([n_{1}]_{p_{n_{1}},q_{n_{1}}}x)=\sum_{k_{1}=0}^{\infty}\frac{([n_{1}]_{p_{n_{1}},q_{n_{1}}}x)^{k_{1}}}{\gamma_{\mu _{1},p_{n_{1}},q_{n_{1}}}(k_{1})}p_{n_{1}}^{\frac{k_{1}(k_{1}-1)}{2}}$ and $e_{\mu_{2},p_{n_{2}},q_{n_{2}}}([n_{2}]_{p_{n_{2}},q_{n_{2}}}y)=\sum_{k_{2}=0}^{\infty}\frac{([n_{2}]_{p_{n_{2}},q_{n_{2}}}y)^{k_{2}}}{\gamma_{\mu_{2},p_{n_{2}},q_{n_{2}}}(k_{2})}p_{n_{2}}^{\frac{k_{2}(k_{2}-1)}{2}}$.

### Lemma 4.1

*Let* $e_{i,j}:\mathbb{R}_{+}^{2}\rightarrow{}[0,\infty)$, $e_{i,j}=(uv)^{ij}, i,j=0,1,2,\dots$, *be the two*-*dimensional test functions*. *Then the* *q*-*bivariate operators defined in* (4.1) *satisfy the following*:

1. $D_{n_{1},n_{2}}^{(p_{n_{1}},p_{n_{2}}),(q_{n_{1}},q_{n_{2}})}(e_{0,0};x,y)=1$,
2. $D_{n_{1},n_{2}}^{(p_{n_{1}},p_{n_{2}}),(q_{n_{1}},q_{n_{2}})}(e_{1,0};x,y)=x$,
3. $D_{n_{1},n_{2}}^{(p_{n_{1}},p_{n_{2}}),(q_{n_{1}},q_{n_{2}})}(e_{0,1};x,y)=y$,
4. $D_{n_{1},n_{2}}^{(p_{n_{1}},p_{n_{2}}),(q_{n_{1}},q_{n_{2}})}(e_{2,0};x,y)\leq x^{2}+\frac{1}{[n_{1}]_{p_{n_{1}},q_{n_{1}}}}[1+2\mu_{1}]_{p_{n_{1}},q_{n_{1}}}x$,
5. $D_{n_{1},n_{2}}^{(p_{n_{1}},p_{n_{2}}),(q_{n_{1}},q_{n_{2}})}(e_{0,2};x,y)\leq y^{2}+\frac{1}{[n_{2}]_{p_{n_{2}},q_{n_{2}}}}[1+2\mu_{2}]_{p_{n_{2}},q_{n_{2}}}y$.

### Lemma 4.2

*The* *q*-*bivariate operators defined in* (4.1) *satisfy the following*:

1. $D_{n_{1},n_{2}}^{(p_{n_{1}},p_{n_{2}}),(q_{n_{1}},q_{n_{2}})}(e_{1,0}-x;x,y)=0$,
2. $D_{n_{1},n_{2}}^{(p_{n_{1}},p_{n_{2}}),(q_{n_{1}},q_{n_{2}})}(e_{0,1}-y;x,y)=0$,
3. $D_{n_{1},n_{2}}^{(p_{n_{1}},p_{n_{2}}),(q_{n_{1}},q_{n_{2}})} ((e_{1,0}-x )^{2};x,y)\leq\frac{1}{[n_{1}]_{p_{n_{1}},q_{n_{1}}}}[1+2\mu_{1}]_{p_{n_{1}},q_{n_{1}}}x$,
4. $D_{n_{1},n_{2}}^{(p_{n_{1}},p_{n_{2}}),(q_{n_{1}},q_{n_{2}})} ( (e_{0,1}-y ) ^{2};x,y)\leq\frac{1}{[n_{2}]_{p_{n_{2}},q_{n_{2}}}}[1+2\mu_{2}]_{p_{n_{2}},q_{n_{2}}}y$.

The rate of convergence of operators $D_{n_{1},n_{2}}^{(p_{n_{1}},p_{n_{2}}),(q_{n_{1}},q_{n_{2}})}(f;x,y)$ defined in (4.1) by means of modulus of continuity of some bivariate modulus of smoothness functions is now introduced.

To obtain the convergence results for the operators $D_{n_{1},n_{2}}^{(p_{n_{1}},p_{n_{2}}),(q_{n_{1}},q_{n_{2}})}(f;x,y)$, we take $q=q_{n_{1}}, q_{n_{2}}$ and $p=p_{n_{1}}, p_{n_{2}}$ where $0< q_{n_{1}}< p_{n_{1}}\leq 1 $ and $0< q_{n_{2}}< p_{n_{2}}\leq1$ are such that

4.2 $$\begin{aligned} \lim_{n_{1},n_{2}}p_{n_{1}}, p_{n_{2}} \to1 \quad\mbox{and}\quad \lim_{n_{1},n_{2}}q_{n_{1}}, q_{n_{2}} \to1. \end{aligned}$$

The modulus of continuity for the bivariate case is defined as follows:

For $f\in H_{\omega}(\mathbb{R}_{+}^{2})$,

4.3 $$\begin{aligned} &\widetilde{\omega}(f;\delta_{1},\delta_{2}) \\ &\quad=\sup_{u,x\geq0} \bigl\{ \bigl\vert f(u,v)-f(x,y) \bigr\vert ; \vert u-x \vert \leq\delta_{1}, \vert v-y \vert \leq\delta_{2}, (u,v), (x,y)\in \mathbb{R}_{+}^{2} \bigr\} , \end{aligned}$$

where $H_{\omega}(\mathbb{R}^{+})$ is the space of all real-valued continuous functions. Then for all $f\in H_{\omega}(\mathbb{R}_{+})$, $\widetilde{\omega}(f;\delta_{1},\delta_{2})$ satisfies the following:

(i) $\lim_{\delta_{1},\delta_{2} \to0}\widetilde{\omega}(f; \delta_{1},\delta_{2})\to0$,
(ii) $\vert f(u,v)-f(x,y) \vert \leq\widetilde{\omega}(f; \delta_{1},\delta_{2}) (\frac{ \vert u-x \vert }{\delta_{1}}+1 ) (\frac{ \vert v-y \vert }{\delta_{2}}+1 )$.

### Theorem 4.3

*Let* $p_{n}=p_{n_{1}},p_{n_{2}}$ *and* $q_{n}=q_{n_{1}},q_{n_{2}}$ *satisfy* (4.2) *and consider* $(x,y) \in[0,\infty), 0< q_{n_{1}},q_{n_{2}}< p_{n_{1}},p_{n_{2}}\leq1$. *Suppose* $D_{n_{1},n_{2}}^{(p_{n_{1}},p_{n_{2}}),(q_{n_{1}},q_{n_{2}})}(f;x,y)$ *are the operators defined by* (4.1). *Then for any function* $f\in\tilde{C} ([0,\infty)\times[0,\infty) )$, *we have*

$$\begin{aligned} &\bigl\vert D_{n_{1},n_{2}}^{(p_{n_{1}},p_{n_{2}}),(q_{n_{1}},q_{n_{2}})}(f;x,y)-f(x,y) \bigr\vert \\ &\quad \leq \bigl\{ 1+\sqrt{[1+2\mu_{1}]_{p_{n_{1}},q_{n_{1}}}x} \bigr\} \bigl\{ 1+\sqrt{ [1+2\mu_{2}]_{p_{n_{2}},q_{n_{2}}}y} \bigr\} \\ &\qquad{}\times \omega \biggl(f;\frac{1}{\sqrt{[n_{1}]_{p_{n_{1}},q_{n_{1}}}}},\frac {1}{\sqrt{[n_{2}]_{p_{n_{2}},q_{n_{2}}}}} \biggr), \end{aligned}$$

*where* $\tilde{C}[0,\infty)$ *is the space of uniformly continuous functions on* $\mathbb{R}^{+}$ *and* $\widetilde{\omega}(f,\delta_{n_{1}},\delta_{n_{2}})$ *is the modulus of continuity of the function* $f \in\tilde{C} ([0,\infty) \times[0,\infty) )$ *defined in* (4.3).

### Proof

We prove the claim by using the results for the modulus of continuity and Cauchy–Schwarz inequality:

$$\begin{aligned} &\bigl\vert D_{n_{1},n_{2}}^{(p_{n_{1}},p_{n_{2}}),(q_{n_{1}},q_{n_{2}})}(f;x,y)-f(x,y) \bigr\vert \\ &\quad\leq \frac{1}{e_{\mu _{1},p_{n_{1}},q_{n_{1}}}([n_{1}]_{p_{n_{1}},q_{n_{1}}}x)}\frac{1}{e_{\mu_{2},p_{n_{2}},q_{n_{2}}}([n_{2}]_{p_{n_{2}},q_{n_{2}}}y)} \\ &\qquad{} \times \sum_{k_{1}=0}^{\infty}\sum _{k_{2}=0}^{\infty}\frac{([n_{1}]_{p_{n_{1}},q_{n_{1}}}x)^{k_{1}}}{\gamma_{\mu _{1},p_{n_{1}},q_{n_{1}}}(k_{1})}\frac{([n_{2}]_{p_{n_{2}},q_{n_{2}}}y)^{k_{2}}}{\gamma_{\mu_{2},p_{n_{2}},q_{n_{2}}}(k_{2})} p_{n_{1}}^{\frac{k_{1}(k_{1}-1)}{2}}p_{n_{2}}^{\frac{k_{2}(k_{2}-1)}{2}} \\ &\qquad{} \times \biggl\vert f \biggl( \frac{p_{n_{1}}^{2\mu_{1}\theta _{k_{1}}+k_{1}}-q_{n_{1}}^{2\mu_{1}\theta_{k_{1}}+k_{1}}}{p_{n_{1}}^{k_{1}-1}(p_{n_{1}}^{n_{1}}-q_{n_{1}}^{n_{1}})}, \frac{p_{n_{2}}^{2\mu_{2}\theta_{k_{2}}+k_{2}}-q_{n_{2}}^{2\mu_{2}\theta_{k_{2}}+k_{2}}}{p_{n_{2}}^{k_{2}-1}(p_{n_{2}}^{n_{2}}-q_{n_{2}}^{n_{2}})} \biggr) -f(x,y) \biggr\vert \\ &\quad\leq \frac{1}{e_{\mu _{1},p_{n_{1}},q_{n_{1}}}([n_{1}]_{p_{n_{1}},q_{n_{1}}}x)}\frac{1}{e_{\mu_{2},p_{n_{2}},q_{n_{2}}}([n_{2}]_{p_{n_{2}},q_{n_{2}}}y)} \\ & \qquad{}\times\sum_{k_{1}=0}^{\infty}\sum _{k_{2}=0}^{\infty}\frac{([n_{1}]_{p_{n_{1}},q_{n_{1}}}x)^{k_{1}}}{\gamma_{\mu _{1},p_{n_{1}},q_{n_{1}}}(k_{1})}\frac{([n_{2}]_{p_{n_{2}},q_{n_{2}}}y)^{k_{2}}}{\gamma_{\mu_{2},p_{n_{2}},q_{n_{2}}}(k_{2})} p_{n_{1}}^{\frac{k_{1}(k_{1}-1)}{2}}p_{n_{2}}^{\frac{k_{2}(k_{2}-1)}{2}} \\ & \qquad{}\times \biggl(1+ \frac{1}{\delta_{n_{1}}} \biggl\vert \biggl( \frac{p_{n_{1}}^{2\mu_{1}\theta_{k_{1}}+k_{1}}-q_{n_{1}}^{2\mu_{1}\theta_{k_{1}}+k_{1}}}{p_{n_{1}}^{k_{1}-1}(p_{n_{1}}^{n_{1}}-q_{n_{1}}^{n_{1}})} \biggr) -x \biggr\vert \biggr) \\ &\qquad{} \times\biggl(1+ \frac{1}{\delta_{n_{2}}} \biggl\vert \biggl( \frac{p_{n_{2}}^{2\mu_{2}\theta_{k_{2}}+k_{2}}-q_{n_{2}}^{2\mu_{2}\theta_{k_{2}}+k_{2}}}{p_{n_{2}}^{k_{2}-1}(p_{n_{2}}^{n_{2}}-q_{n_{2}}^{n_{2}})} \biggr) -y \biggr\vert \biggr)\widetilde{ \omega}(f;\delta_{n_{1}},\delta_{n_{2}}) \\ &\quad\leq \Biggl\{ 1+\frac{1}{\delta_{n_{1}}} \Biggl( \frac{1}{e_{\mu_{1},p_{n_{1}},q_{n_{1}}}([n_{1}]_{p_{n_{1}},q_{n_{1}}}x)}\sum _{k_{1}=0}^{\infty}\frac{([n_{1}]_{p_{n_{1}},q_{n_{1}}}x)^{k_{1}}}{\gamma_{\mu_{1},p_{n_{1}},q_{n_{1}}}(k_{1})}p_{n_{1}}^{\frac{k_{1}(k_{1}-1)}{2}}\\ &\qquad{}\times \biggl( \frac{p_{n_{1}}^{2\mu_{1}\theta_{k_{1}}+k_{1}}-q_{n_{1}}^{2\mu_{1}\theta_{k_{1}}+k_{1}}}{p_{n_{1}}^{k_{1}-1}(p_{n_{1}}^{n_{1}}-q_{n_{1}}^{n_{1}})}-x \biggr)^{2} \Biggr)^{\frac {1}{2}} \Biggr\} \\ &\qquad {}\times \Biggl\{ 1+\frac{1}{\delta_{n_{2}}} \Biggl( \frac{1}{e_{\mu_{2},p_{n_{2}},q_{n_{2}}}([n_{2}]_{p_{n_{2}},q_{n_{2}}}y)}\sum _{k_{2}=0}^{\infty}\frac{([n_{2}]_{p_{n_{2}},q_{n_{2}}}y)^{k_{2}}}{\gamma_{\mu _{2},p_{n_{2}},q_{n_{2}}}(k_{2})}p_{n_{2}}^{\frac{k_{2}(k_{2}-1)}{2}}\\ &\qquad{}\times \biggl( \frac{p_{n_{2}}^{2\mu_{2}\theta_{k_{2}}+k_{2}}-q_{n_{2}}^{2\mu_{2}\theta_{k_{2}}+k_{2}}}{p_{n_{2}}^{k_{2}-1}(p_{n_{2}}^{n_{2}}-q_{n_{2}}^{n_{2}})}-y \biggr)^{2} \Biggr)^{\frac {1}{2}} \Biggr\} \\ &\qquad{} \times \widetilde{\omega}(f;\delta_{n_{1}},\delta_{n_{2}}) \\ &\quad = \biggl(1+\frac{1}{\delta_{n_{1}}} \bigl(D_{n_{1},n_{2}}^{(p_{n_{1}},p_{n_{2}}),(q_{n_{1}},q_{n_{2}})}(e_{1,0}-x)^{2};x,y \bigr)^{\frac{1}{2}} \biggr) \\ &\qquad{}\times\biggl(1+\frac{1}{\delta_{n_{2}}} \bigl(D_{n_{1},n_{2}}^{(p_{n_{1}},p_{n_{2}}),(q_{n_{1}},q_{n_{2}})}(e_{0,1}-y)^{2};x,y \bigr)^{\frac{1}{2}} \biggr) \\ &\qquad {}\times \widetilde{\omega}(f;\delta_{n_{1}},\delta_{n_{2}}) \\ &\quad \leq \biggl(1+\frac{1}{\delta_{n_{1}}} \sqrt{\frac {1}{[n_{1}]_{p_{n_{1}},q_{n_{1}}}}[1+2 \mu_{1}]_{p_{n_{1}},q_{n_{1}}}x} \biggr) \\ &\qquad{} \times \biggl(1+\frac{1}{\delta_{n_{2}}} \sqrt{\frac{1}{[n_{2}]_{p_{n_{2}},q_{n_{2}}}}[1+2 \mu_{2}]_{p_{n_{2}},q_{n_{2}}}y} \biggr) \widetilde {\omega}(f; \delta_{1},\delta_{2}). \end{aligned}$$

Choosing $\delta_{1}=\delta_{n_{1}}=\sqrt{\frac{1}{[n_{1}]_{q_{n_{1}}}}}$ and $\delta_{2}=\delta_{n_{2}}=\sqrt{\frac{1}{[n_{2}]_{q_{n_{2}}}}}$ yields our result. □

Now we give the rate of convergence of the operators $D_{n_{1},n_{2}}^{(p_{n_{1}},p_{n_{2}}),(q_{n_{1}},q_{n_{2}})}(f;x,y)$ defined in (4.1) in terms of the elements of the usual Lipschitz class $\operatorname{Lip}_{M}(\nu_{1},\nu_{2} )$.

Let $f\in C([0,\infty)\times[0,\infty)) $, $M>0$ and $0<\nu_{1},\nu_{2} \leq1$. We recall that the class $\operatorname{Lip}_{M}(\nu_{1},\nu_{2} )$ is defined by

4.4 $$ \operatorname{Lip}_{M}(\nu_{1},\nu_{2} )= \bigl\{ f: \bigl\vert f(u,v)-f(x,y) \bigr\vert \leq M \vert u-x \vert ^{\nu_{1} } \vert v-y \vert ^{\nu_{2} }, (u,v), (x,y)\in [0, \infty)^{2} \bigr\} . $$

### Theorem 4.4

*Let* $D_{n_{1},n_{2}}^{(p_{n_{1}},p_{n_{2}}),(q_{n_{1}},q_{n_{2}})}(f;x,y)$ *be the operator defined in* (4.1). *Then for each* $f\in \operatorname{Lip}_{M}(\nu_{1},\nu_{2} ), M>0, 0<\nu_{1},\nu_{2} \leq1$, *satisfying* (4.4), *we have*

$$ \bigl\vert D_{n_{1},n_{2}}^{(p_{n_{1}},p_{n_{2}}),(q_{n_{1}},q_{n_{2}})}(f;x,y)-f(x,y) \bigr\vert \leq M \bigl(\lambda_{n_{1}}(x) \bigr)^{\frac{\nu_{1}}{2}} \bigl( \lambda_{n_{2}}(y) \bigr)^{\frac{\nu_{2}}{2}}, $$

*where* $\lambda _{n_{1}}(x)=D_{n_{1},n_{2}}^{(p_{n_{1}},p_{n_{2}}),(q_{n_{1}},q_{n_{2}})} ((e_{1,0}-x)^{2};x,y )$, *and* $\lambda_{n_{2}}(y)=D_{n_{1},n_{2}}^{(p_{n_{1}},p_{n_{2}}),(q_{n_{1}},q_{n_{2}})} ((e_{0,1}-y)^{2};x,y )$.

### Proof

We prove the claim by using (4.4) and Hölder inequality:

$$\begin{aligned} &\bigl\vert D_{n_{1},n_{2}}^{(p_{n_{1}},p_{n_{2}}),(q_{n_{1}},q_{n_{2}})}(f;x,y)-f(x,y) \bigr\vert \\ &\quad\leq \bigl\vert D_{n_{1},n_{2}}^{(p_{n_{1}},p_{n_{2}}),(q_{n_{1}},q_{n_{2}})}\bigl(f(u,v)-f(x,y);x,y \bigr) \bigr\vert \\ &\quad\leq D_{n_{1},n_{2}}^{(p_{n_{1}},p_{n_{2}}),(q_{n_{1}},q_{n_{2}})} \bigl( \bigl\vert f(u,v)-f(x,y) \bigr\vert ;x,y \bigr) \\ &\quad \leq \bigl\vert D_{n_{1},n_{2}}^{(p_{n_{1}},p_{n_{2}}),(q_{n_{1}},q_{n_{2}})} \bigl( \vert e_{1,0}-x \vert ^{\nu_{1}};x,y \bigr) \bigr\vert D_{n_{1},n_{2}}^{(p_{n_{1}},p_{n_{2}}),(q_{n_{1}},q_{n_{2}})} \bigl( \vert e_{0,1}-y \vert ^{\nu_{2}};x,y \bigr). \end{aligned}$$

Therefore,

$$\begin{aligned} &\bigl\vert D_{n_{1},n_{2}}^{(p_{n_{1}},p_{n_{2}}),(q_{n_{1}},q_{n_{2}})}(f;x,y)-f(x,y) \bigr\vert \\ &\quad \leq M\frac{1}{e_{\mu _{1},p_{n_{1}},q_{n_{1}}}([n_{1}]_{p_{n_{1}},q_{n_{1}}}x)}\sum_{k_{1}=0}^{\infty} \frac{([n_{1}]_{p_{n_{1}},q_{n_{1}}}x)^{k_{1}}}{ \gamma_{\mu_{1},p_{n_{1}},q_{n_{1}}}(k_{1})}p_{n_{1}}^{\frac {k_{1}(k_{1}-1)}{2}} \biggl\vert \frac{p_{n_{1}}^{2\mu_{1}\theta _{k_{1}}+k_{1}}-q_{n_{1}}^{2\mu_{1}\theta_{k_{1}}+k_{1}}}{p_{n_{1}}^{k_{1}-1}(p_{n_{1}}^{n_{1}}-q_{n_{1}}^{n_{1}})}-x \biggr\vert ^{\nu _{1}} \\ &\qquad{}\times \frac{1}{e_{\mu _{2},p_{n_{2}},q_{n_{2}}}([n_{2}]_{p_{n_{2}},q_{n_{2}}}y)}\sum_{k_{2}=0}^{\infty} \frac{([n_{2}]_{p_{n_{2}},q_{n_{2}}}y)^{k_{2}}}{ \gamma_{\mu_{2},p_{n_{2}},q_{n_{2}}}(k_{2})}p_{n_{2}}^{\frac {k_{2}(k_{2}-1)}{2}} \biggl\vert \frac{p_{n_{2}}^{2\mu_{2}\theta _{k_{2}}+k_{2}}-q_{n_{2}}^{2\mu_{2}\theta_{k_{2}}+k_{2}}}{p_{n_{2}}^{k_{2}-1}(p_{n_{2}}^{n_{2}}-q_{n_{2}}^{n_{2}})}-y \biggr\vert ^{\nu _{2}} \\ &\quad \leq M \Biggl( \frac{1}{ ( e_{\mu _{1},p_{n_{1}},q_{n_{1}}}([n_{1}]_{p_{n_{1}},q_{n_{1}}}x) ) }\sum_{k_{1}=0}^{\infty} \frac{([n_{1}]_{p_{n_{1}},q_{n_{1}}}x)^{k_{1}}p_{n_{1}}^{\frac {k_{1}(k_{1}-1)}{2}}}{\gamma_{\mu_{1},p_{n_{1}},q_{n_{1}}}(k_{1})} \Biggr) ^{\frac{2-\nu _{1}}{2}} \\ &\qquad{}\times \Biggl( \frac{1}{ ( e_{\mu _{1},p_{n_{1}},q_{n_{1}}}([n_{1}]_{p_{n_{1}},q_{n_{1}}}x) ) }\\ &\qquad{}\times \sum _{k_{1}=0}^{\infty}\frac{([n_{1}]_{p_{n_{1}},q_{n_{1}}}x)^{k_{1}}p_{n_{1}}^{\frac {k_{1}(k_{1}-1)}{2}}}{\gamma_{\mu_{1},p_{n_{1}},q_{n_{1}}}(k_{1})} \biggl\vert \frac{p_{n_{1}}^{2\mu_{1}\theta_{k_{1}}+k_{1}}-q_{n_{1}}^{2\mu_{1}\theta _{k_{1}}+k_{1}}}{p_{n_{1}}^{k_{1}-1}(p_{n_{1}}^{n_{1}}-q_{n_{1}}^{n_{1}})}-x \biggr\vert ^{2} \Biggr) ^{\frac{\nu_{1}}{2}} \\ &\qquad{}\times \Biggl( \frac{1}{ ( e_{\mu _{2},p_{n_{2}},q_{n_{2}}}([n_{2}]_{p_{n_{2}},q_{n_{2}}}y) ) }\sum _{k_{2}=0}^{\infty}\frac{([n_{2}]_{p_{n_{2}},q_{n_{2}}}y)^{k_{2}}p_{n_{2}}^{\frac {k_{2}(k_{2}-1)}{2}}}{\gamma_{\mu_{2},p_{n_{2}},q_{n_{2}}}(k_{2})} \Biggr) ^{\frac{2-\nu _{2}}{2}} \\ &\qquad{}\times \Biggl( \frac{1}{ ( e_{\mu _{2},p_{n_{2}},q_{n_{2}}}([n_{2}]_{p_{n_{2}},q_{n_{2}}}y) ) }\\ &\qquad{}\times \sum _{k_{2}=0}^{\infty}\frac{([n_{2}]_{p_{n_{2}},q_{n_{2}}}y)^{k_{2}}p_{n_{2}}^{\frac {k_{2}(k_{2}-1)}{2}}}{\gamma_{\mu_{2},p_{n_{2}},q_{n_{2}}}(k_{2})} \biggl\vert \frac{p_{n_{2}}^{2\mu_{2}\theta_{k_{2}}+k_{2}}-q_{n_{2}}^{2\mu_{2}\theta _{k_{2}}+k_{2}}}{p_{n_{2}}^{k_{2}-1}(p_{n_{2}}^{n_{2}}-q_{n_{2}}^{n_{2}})}-y \biggr\vert ^{2} \Biggr) ^{\frac{\nu_{2}}{2}} \\ &\quad\leq \bigl( D_{n_{1},n_{2}}^{(p_{n_{1}},p_{n_{2}}),(q_{n_{1}},q_{n_{2}})}(e_{1,0}-x)^{2};x,y \bigr) ^{\frac{\nu_{1}}{2}} \bigl( D_{n_{1},n_{2}}^{(p_{n_{1}},p_{n_{2}}),(q_{n_{1}},q_{n_{2}})}(e_{0,1}-y)^{2};x,y \bigr) ^{\frac{\nu_{2}}{2}}. \end{aligned}$$

This completes the proof. □

## Conclusion

In this paper we have constructed a $(p,q)$-analogue of Dunkl type Szász operators. We obtained some approximation results for these operators and showed the convergence of the operators by using the modulus of continuity. Furthermore, we obtained the rate of convergence of these operators for functions belonging to the Lipschitz class. We have also studied the bivariate version of these operators.
